# Selective targeting of *KRAS* oncogenic alleles by CRISPR/Cas9 inhibits proliferation of cancer cells

**DOI:** 10.1038/s41598-018-30205-2

**Published:** 2018-08-08

**Authors:** Wookjae Lee, Joon Ho Lee, Soyeong Jun, Ji Hyun Lee, Duhee Bang

**Affiliations:** 1 Department of Chemistry, Yonsei University, Seoul, Korea; 2 Department of Clinical Pharmacology and Therapeutics, College of Medicine, Kyung Hee University, Seoul, Korea; 3 Department of Biomedical Science and Technology, Kyung Hee Medical Science Research Institute, Kyung Hee University, Seoul, Korea

## Abstract

Mutations within the *KRAS* oncogene are associated with the proliferation of various cancers. Therapeutic approaches for treating cancers with such mutations have focused on targeting the downstream protein effectors of KRAS. However, to date, no approved treatment has targeted the mutated *KRAS* oncogene directly. Presently, we used the selectivity of the CRISPR/Cas9 system to directly target mutated *KRAS* alleles. We designed single-guide RNAs (sgRNAs) to target two specific single-nucleotide missense mutations on *KRAS* codon-12 located in the seed region adjacent to a protospacer adjacent motif (PAM). Lentiviral transduction of Cas9 and the sgRNAs into cancer cells with respective *KRAS* mutations resulted in high frequency of indels in the seed region. Indel-associated disruption of the mutant *KRAS* alleles correlated with reduced viability of the cancer cells. The results indicate that CRISPR-Cas9-mediated genome editing can potentially be used for the treatment of cancer patients, specifically those with oncogenic *KRAS* mutations.

## Introduction

Combined global effort has identified over 600 cancer-inducing somatic mutations that have been catalogued online (COSMIC v82 database). Mutated *RAS* genes, among the first identified oncogenes, were initially discovered in human cancers in the 1980s^1,2^. Oncogenic *RAS* mutations contribute to the induction of various cancers including pancreatic ductal adenocarcinomas (PDACs)^3–5^, colorectal adenocarcinomas (CRCs)^6–9^, lung adenocarcinomas^10–13^, and gastric adenocarcinomas^14,15^. Mutated *RAS* genes, representing the most frequently mutated oncogene family, are present in approximately 25% of human tumors^16^. Mutations within *KRAS* account for 85% of all *RAS* family oncogenic mutations^16^. A common cancer-associated mutation occurs in *KRAS* at the glycine-encoding codon-12. Specifically, the single-nucleotide missense substitutions c.35 G > T and c.35 G > A replace glycine at position 12 with valine (G12V) and aspartic acid (G12D), respectively. G12V and G12D substitutions are among the most commonly observed mutations in pancreatic adenocarcinoma (30% and 51%, respectively) and colorectal adenocarcinomas (27% and 45%, respectively) and have been associated with poor prognosis^2^. Thus, specific therapeutic approaches are needed to treat patients with *KRAS* codon-12 oncogenic mutations.

Various attempts have been made to target RAS-dependent cancers^2,16,17^. Among them, approaches aimed at targeting the downstream effectors of mutated RAS have led to the development and approval of several chemotherapeutic agents, including regorafenib, sorafenib, selumetinib, and trametinib^2^. Although directly targeting mutated *RAS* oncogenes has the potential to disrupt the functions of both the aberrant RAS proteins and their downstream effector pathways, the direct inhibition of RAS proteins has not been successful. Due to the difficulties of targeting protein directly, a recent paper showed that mutated *KRAS* gene can be targeted with a synthetic alkylating agent of pyrrole–imidazole polyamide indole-*seco*-CBI conjugate^18^. However, producing this chemical is challenging due to its complex structure. Hence, alternative approaches for specific targeting of the mutated *KRAS* genes are needed.

The prokaryotic clustered regularly interspaced short palindromic repeats (CRISPR)/CRISPR associated protein 9 (Cas9) system from *Streptococcus pyogenes* has been successfully used to edit the genomes of mammalian cells using a chimeric single-guide RNA (sgRNA)^19–24^. The sgRNA directs the Cas9 nuclease to a complementary target sequence that also contains a protospacer adjacent motif (PAM), with the PAM-proximal bases known as the seed region^25^. Directed by the sgRNA, the Cas9 introduces DNA double-strand breaks (DSBs) in the target DNA, which may undergo non-homologous end-joining (NHEJ)^26^. Correction of DSBs via error-prone NHEJ commonly results in random insertions or deletions (indels), potentially rendering the target gene nonfunctional. Such disruption of target genes may be beneficial for targeting oncogenes.

In this study, we developed a method to impair cancer cell proliferation by directly targeting two specific mutations at codon-12 of the *KRAS* oncogene. The single-nucleotide missense substitutions c.35 G > T and c.35 G > A, resulting in G12V and G12D, respectively, were selected to take advantage of a neighboring PAM sequence (5′-NGG-3′) within the seed region. We reasoned that the CRISPR/Cas9 system would selectively target and disrupt the oncogenic alleles, leading to inhibition of cancer cell growth. As expected, *KRAS* mutant-specific CRISPR/Cas9 selectively inhibited the growth of various cancer cell lines with *KRAS* mutations *in vitro*.

## Results

### Designing a sgRNA to target a single-nucleotide substitution on codon-12 of *KRAS*

**Figure 1:**
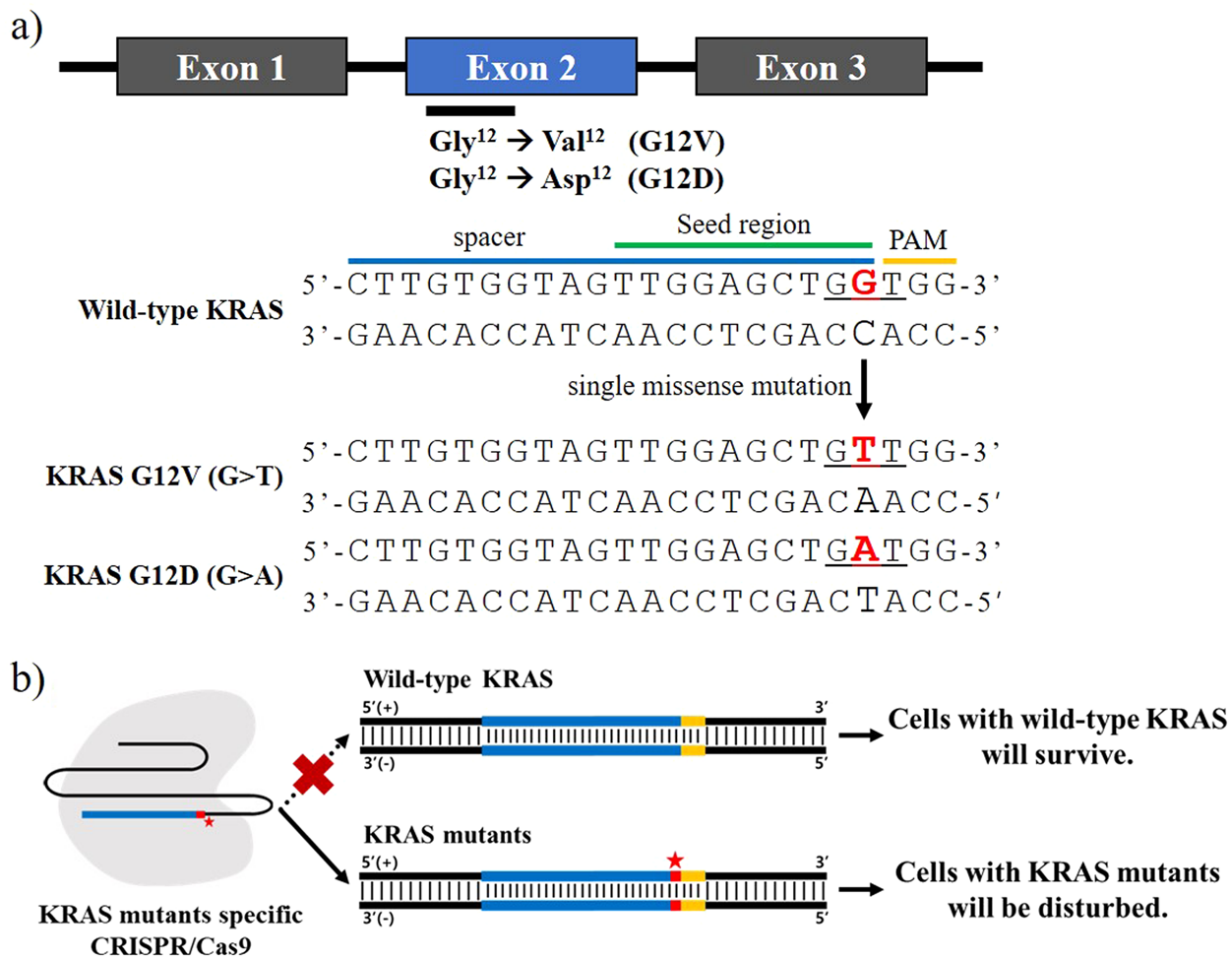
Rationale for CRISPR/Cas9-based disruption of cancer cells with oncogenic *KRAS* mutations. (**a**) Location of oncogenic *KRAS* single-nucleotide substitutions (c.35 G > T and c.35 G > A) resulting in G12V and G12D substitutions, and the designs of sgRNAs. Mutations are located within exon-2 of *KRAS*. To design the sgRNAs, the two single missense mutations in the seed region within a spacer adjacent to the PAM sequence were selected. The positions of the single-nucleotide substitutions are indicated in red. (**b**) The CRISPR/Cas9 system was designed to selectively target and inhibits the proliferation of cancer cells with mutant *KRAS* alleles. Blue strands: spacer; yellow strands: PAM sequence; red strands and star: single-nucleotide missense mutations.

We sought to directly target oncogenic *KRAS* using the CRISPR/Cas9 system, and consequently impair the growth of cancer cells. Major oncogenic mutations occur on codon-12 of *KRAS* exon-2 (Fig. 1a). The single-nucleotide missense substitution c.35 G > T changes the encoded glycine to a valine (G12V; Fig. 1a). This mutated target nucleotide is located within the seed region adjacent to the PAM sequence, and was thus chosen to be targeted by CRISPR/Cas9. We designed a sgRNA to target the *KRAS* c.35 G > T mutant allele (designated as sgKRAS-G12V). We also designed a sgRNA to target the wild-type KRAS (sgKRAS-WT) as a negative control for the cell lines with the *KRAS* G12V mutation. We then separately incorporated sgKRAS-WT and sgKRAS-G12V into the lentiviral backbone plasmid lentiCRISPR v2 encoding Cas9. We hypothesized that the *KRAS* mutant-specific CRISPR/Cas9 system will selectively target the mutant alleles, therefore exclusively affecting cell lines with the respective *KRAS* mutations, but not cell lines with wild-type alleles (Fig. 1b).

### Selective targeting of *KRAS* c.35 G > T by CRISPR/Cas9 in CRC cell lines

**Figure 2:**
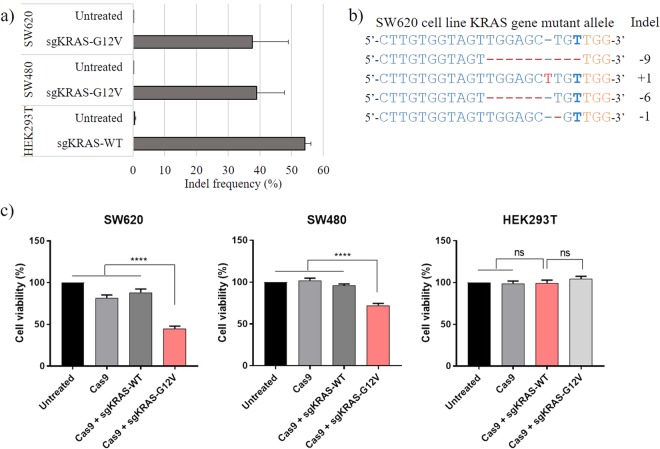
CRISPR/Cas9 targeting of *KRAS* c.35 G > T in SW620 and SW480 cells. (**a**) Indel frequencies in target *KRAS* region of SW620 and SW480 cells with sgKRAS-G12V, and in HEK293T cells with sgKRAS-WT. (**b**) Major indel patterns detected in the *KRAS* target region of SW620 cells with sgKRAS-G12V. Blue: sgRNA target sequence; bold blue: c.35 G > T single-nucleotide missense substitution; orange: PAM sequence; red: mismatched nucleotides. (**c**) Cell proliferation assays of indicated cell lines following transduction with lentiviral vector encoding Cas9, Cas9 and sgKRAS-G12V, or Cas9 and sgKRAS-WT. Red: sgRNA sample relevant to tested cell line. Bars represent the mean ± S.E.M. ^****^(P < 0.0001), ns: not significant.

We selected two CRC cell lines, SW620 and SW480, containing the *KRAS* c.35 G > T (G12V) mutant allele (Supplementary Table 1). Due to the lack of the corresponding *KRAS* mutation, HEK293T cells were selected as a control. To test whether the designed sgRNAs are functioning properly, we transduced the experimental cell lines (SW620 and SW480) and the control cell line (HEK293T) with the lentiviral vectors expressing Cas9 and the two sgRNAs (sgKRAS-G12V and sgKRAS-WT). We performed two additional experiments as controls to validate our results. One of the controls was without any lentiviral treatment, and the other was a lentiviral vector which only expressed Cas9 while lacking sgRNA. Subsequently, we used next-generation sequencing (NGS) to analyze the target *KRAS* locus of each cell line. SW620 and SW480 cells with sgKRAS-G12V contained indels in the target region at frequencies of 37.7% and 38.95%, respectively (Fig. 2a,b, Supplementary Fig. 1a). We also observed a 54.2% indel frequency in HEK293T cells with sgRNA targeting wild-type *KRAS* (Fig. 2a, Supplementary Fig. 1b). Additionally, we observed insignificant indel frequencies (less than 1%) when the *KRAS* sequence of cell lines did not match the proper sgRNAs, such as SW620 cells with sgKRAS-WT (Supplementary Fig. 2a). However, when the *KRAS* sequence of each cell line was complementary to the sgRNA, we observed indel patterns (Supplementary Fig. 1c~e), and these indels caused frameshifts exceeding 50%. Based on the results, we hypothesized that if indels and frameshifts of *KRAS* occur due to the specific sgRNAs, this would affect cell viability of cancer cells with *KRAS* mutations. Hence, we subsequently performed cell viability assays to confirm our hypothesis that cancer cell lines with specific mutations can be targeted using the CRISPR/Cas9 system.

### CRC cell viability following the targeting of oncogenic *KRAS* c.35 G > T mutation by CRISPR/Cas9

We transduced SW620, SW480, and HEK293T cell lines with Cas9-expressing lentiviral vectors with sgKRAS-G12V or sgKRAS-WT. Cells transduced with lentiviral vectors with Cas9 in the absence of sgRNAs were used as negative controls. Three days following the procedure, the viability of the transduced cells was assessed using 3-(4,5-dimethylthiazol-2-yl)-2, 5-diphenyltetrazolium bromide (MTT) cell proliferation assays (Fig. 2c). The viability of cells with sgKRAS-WT or with Cas9 alone was similar to that of untreated cells. In contrast, the viability of SW620 and SW480 cells transduced with sgRNA targeting *KRAS* c.35 G > T was reduced to 44.8% and 72.2%, respectively. In contrast, none of the treatments significantly affected the growth of HEK293T cells. The MTT assay indicated that specifically targeting the *KRAS* c.35 G > T mutant allele negatively affected the growth of SW620 and SW480 CRC cell lines. Taken together, the NGS and MTT assay results suggested that the Cas9 nuclease can be used to distinguish between specific *KRAS* oncogene variants and used to selectively inhibit the growth of specific cancer cell lines.

### Inducing disruption of cancer cells harboring the KRAS G12D variant

Following the successful targeting of *KRAS* c.35 G > T by the CRSPR-Cas9 system (Fig. 1), we hypothesized that cancer cell lines harboring the KRAS G12D variant can likewise be targeted. The KRAS G12D variant, with position-12 glycine substituted to an aspartic acid, similarly arises from the single-nucleotide missense mutation c.35 G > A (Fig. 1a). Hence, we designed a new sgRNA to target *KRAS* c.35 G > A (generating sgKRAS-G12D). As in the previous experiment, sgRNA targeting wild-type *KRAS* (sgKRAS-WT) was used as a control.

**Figure 3:**
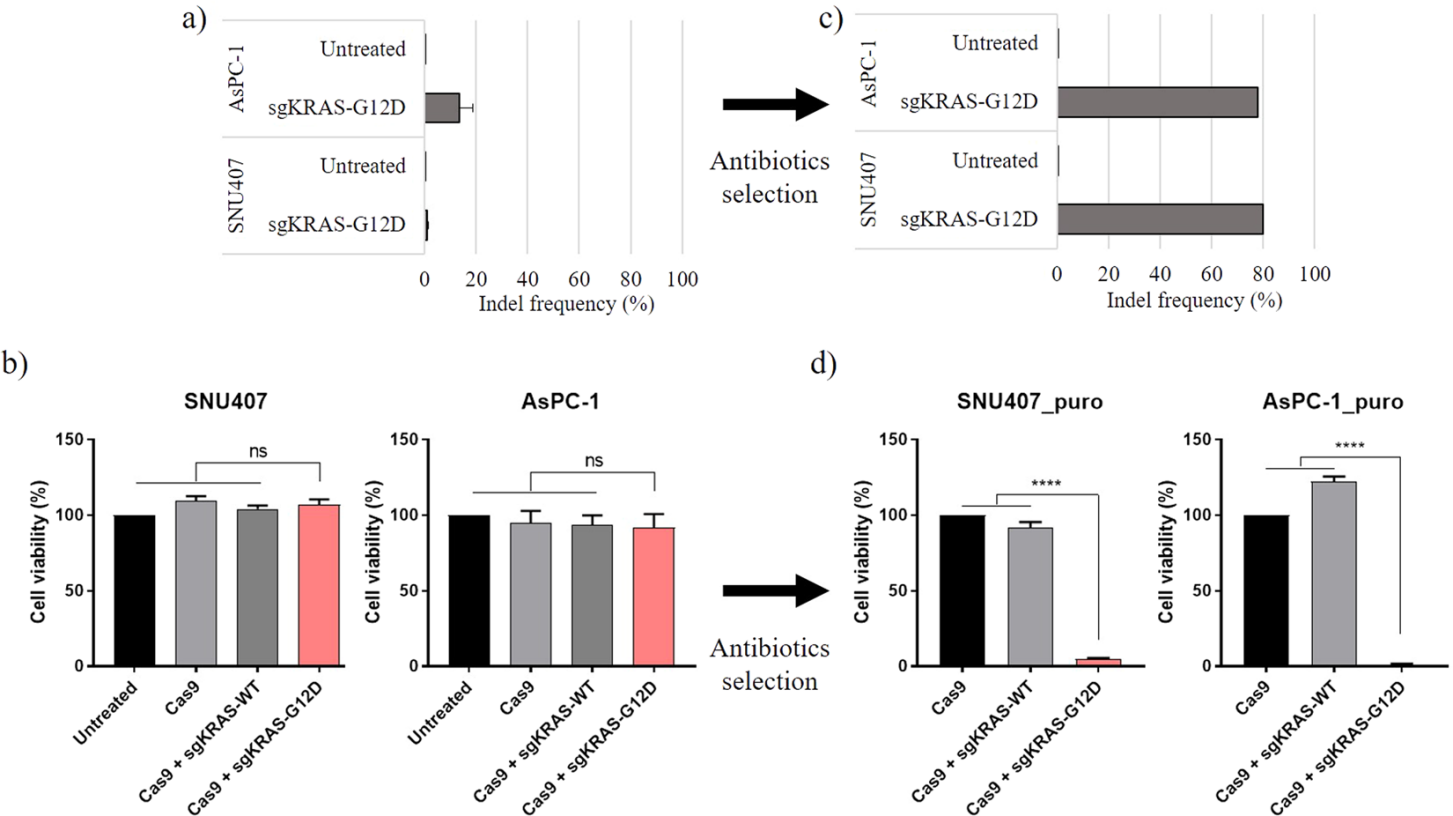
Indel ratios and transduced cell viability before and after puromycin selection. Indel frequencies in target *KRAS* region of AsPC-1 and SNU407 cells with sgKRAS-G12D were determined before (**a**) and after (**c**) antibiotic selection. Transduced cell viability was determined using the MTT assay before (**b**) and after (**d**) puromycin selection. For cell proliferation assays, AsPC-1 and SNU407 cells were transfected with lentiviral vectors encoding Cas9, Cas9 and sgKRAS-G12D, or Cas9 and sgKRAS-WT. Red: sgRNA sample relevant to tested cell line. Bars represent the mean ± S.E.M. ^****^(P < 0.0001), ns: not significant.

We selected two cell lines, AsPC-1 SNU407, derived from patients with PDAC and CRC, respectively, containing the *KRAS* c.35 G > A mutant allele (Supplementary Table 1). These cell lines were transduced with lentiviral vectors with or without sgRNA targeting wild-type or c.35 G > A *KRAS* alleles. HEK293T was used as a control cell line. Viability of transduced cells was assessed using the MTT cell proliferation assay three days post-transduction (Fig. 3b). However, in contrast to the results from the previous experiment targeting *KRAS* c.35 G > T, the MTT assay data indicated that viability of AsPC-1 and SNU407 cells was not affected by any of the treatments. Sequencing of *KRAS* DNA revealed significantly lower frequencies of indels in AsPC-1 and SNU407 cells (13.45%, 0.95%, respectively) with sgKRAS-G12D than in SW620 and SW480 with sgKRAS-G12V (Figs 2a and 3a). As previous experiments, we observed specificity of sgRNA in indel patterns and frameshifts due to the indels (Supplementary Figs 2 and 3). We hypothesized that these low indel frequencies were caused by cell line-specific reduction in efficiency of lentiviral transductions.

To overcome the limitation of low transduction efficiency, we exploited puromycin resistance imbedded in all lentiviral vectors of testing groups. Because of the puromycin resistance of the vectors, we hypothesized that successfully transfected cells would survive. Thus, puromycin ensured specific selection and analysis of cells containing the desired sgRNAs and the CRISPR system. The selection was performed two days after the lentiviral transduction of AsPC-1 and SNU407 cells. We observed cells from the untreated control group did not survive after applying puromycin. Hence, we utilized the control with only Cas9 without sgRNAs to validate our results. The MTT assays were performed seven days after the start of antibiotic selection in accord with common protocols for mammalian cell lines. Following puromycin selection, cell lines transduced with sgKRAS-G12D exhibited significantly reduced viability (Fig. 3d). The puromycin-selected AsPC-1 and SNU407 cells transduced with *KRAS* c.35 G > A-targeting sgRNA demonstrated significantly increased indel rates of 77.9% and 80.1%, respectively (Fig. 3c). We concluded that the CRISPR-Cas9 targeting of cell lines with the KRAS G12D mutation can be enhanced with antibiotic selection. We confirmed this trend by testing puromycin selection following the transduction of SW620 and SW480 cells with the sgKRAS-G12V CRISPR-Cas9 vector. While targeting of *KRAS* c.35 G > T reduced the viability of SW620 and SW480 cells to 50–70% in the absence of puromycin, antibiotic selection resulted in a further reduction of CRC cell viability to below 5% (Fig. 1c, Supplementary Fig. 4).

## Discussion

The present study demonstrated a novel method for inhibiting the growth of cancer cell lines with oncogenic *KRAS* by targeting the mutated gene using CRISPR/Cas9. We sought to use the CRISPR/Cas9 system to introduce indel mutations into the genomes of cancer cells specifically at the *KRAS* mutated codon-12 sites. We reasoned that because mutated KRAS is crucial for cancer proliferation pathways, indels introduced into the gene via error-prone NHEJ would be detrimental to the survival of the cancer cells. The results of the MTT cell viability assays validated the use of specific sgRNAs to inhibit the proliferation of cancer cells by selectively targeting oncogenic mutations of *KRAS* codon-12.

Differences in transduction efficiencies of the tested cell lines prompted us to utilize puromycin selection to specifically isolate transduced cells. For CRISPR/Cas9 systems targeting both *KRAS* c.35 G > A and *KRAS* c.35 G > T, the puromycin selection resulted in greater inhibition of cancer cell viability. In the absence of puromycin selection, targeting of *KRAS* c.35 G > T reduced the viability of SW620 and SW480 to approximately 50% and 70%, respectively, while targeting of *KRAS* c.35 G > A in AsPC-1 and SNU407 had no effect on viability. In contrast, the puromycin selection procedure reduced viability of all cancer cells lines following the CRISPR/Cas9-mediated targeting of the respective *KRAS* mutations to less than 10%. These results demonstrated that CRISPR/Cas9-mediated inhibition of cancer cell growth is dependent on efficient target cell transduction. Although antibiotic selection may be unfeasible in the clinical setting, improved methods for CRISPR system delivery can help target and inhibit proliferation of cancer cells *in vivo*.

We additionally performed immunoblotting assays to observe changes in protein levels of KRAS after specific sgRNAs and Cas9 were introduced to the cells. We performed the assay with SW620 as an experimental group due to its significant cell proliferation decrease from the previous experiments, and HEK293T as a control to validate our results. After the transduction, we performed puromycin selection to those cell lines following the identical protocol as previous experiments. The result of immunoblotting assays indicated a decreased protein level of KRAS from SW620 cells with sgKRAS-G12V. The assay also indicated a diminished, although weak, protein level from HEK293T with sgKRAS-WT (Supplementary Fig. 5). Combining the results of immunoblotting assays and the previous cell proliferation assays (Fig. 2), SW620 which has an importance in *KRAS* mutations showed decreased results from both protein and cell proliferation assays. However, HEK293T which lacks an importance to the gene only showed decrease in protein levels.

A recent study has similarly provided evidence for the use of the CRISPR/Cas9 system to selectively target an oncogenic mutant allele. Specifically, the authors targeted a single-nucleotide missense mutation in *EGFR* that generated a *S*. *pyogenes* Cas9-recognized PAM. The approach resulted in the killing of cancer cells with the mutation *in vitro* and in the reduction of tumor size in a xenograft mouse model^27^. In contrast to this work, we observed significant inhibition of cancer cell proliferation after targeting mutated sequences within the seed region, rather than within the PAM. Hence, we suggest that targeting oncogenic mutations within either the seed region or the PAM sequence can cause inhibition of cancer cell growth.

We expect that the method described here can be extended further to selectively target other vital oncogenes. Presently, we used the *S*. *pyogenes* orthologue of Cas9 for targeting of oncogenic loci. However, other orthologues of Cas9 can be used to increase the diversity of target PAM sequences, thus allowing to selectively target and disrupt additional oncogenes in various cancers. Although the targeted genome was different, the aforementioned journal showed successful results *in vivo* using the similar technique^27^. Hence, if there is a method to introduce mutation-specific CRISPR system with high efficiency to tumors, we believe halting or reducing cancer proliferation *in vivo* could be possible. Thus, the method described here represents a promising approach for novel directions in cancer therapy.

## Materials and Methods

### Cell lines and cell culture

All cell lines were obtained from KCLB (Korean Cell Line Bank) and maintained at 37 °C with 5% CO_2_. The human embryonic kidney HEK293T cell line was cultured in Dulbecco’s modified Eagle’s medium (DMEM; Gibco, USA) supplemented with 10% fetal bovine serum (FBS) (Gibco, USA) and 1% penicillin/streptomycin (Thermo Fisher Scientific, USA). The human colorectal adenocarcinoma SW480, SW620, and SNU407 cells lines and the pancreatic ductal adenocarcinoma AsPC-1 cell line were cultured in Roswell Park Memorial Institute medium supplemented with 10% FBS and 1% penicillin/streptomycin.

### Lentiviral plasmid construction

The lentiviral plasmid lentiCRISPR v2 was a gift from Feng Zhang (Addgene plasmid #52961, Addgene, USA)^28^. The targeting sgRNA sequences were cloned according to the provided lentiCRISPR v2 cloning protocol. The 20-bp *KRAS* target sequences of the sgRNAs cloned into lentiCRISPR v2 were as follows: wild-type *KRAS*, CTTGTGGTAGTTGGAGCTGG (generating sgKRAS-WT); *KRAS*.c.35 G > T, CTTGTGGTAGTTGGAGCTGT (sgKRAS-G12V); *KRAS*.c.35 G > A, CTTGTGGTAGTTGGAGCTGA (sgKRAS-G12D). Plasmids were prepared using the EndoFree Plasmid Maxi Kit (QIAGEN, USA) and Exprep Plasmid SV kit (GeneAll Biotechnology, Korea) according to manufacturers’ protocols.

### Lentivirus production and titration

HEK293T cells were seeded at 80% confluency in 6-well plates coated with 0.02 mg/mL poly-D-lysine (Sigma-Aldrich, USA). One day after seeding, the cells were transfected in Gibco™ Opti-MEM™ I Reduced Serum Medium (Life Technologies, USA) using 1 μg of target lentiviral vector (empty lentiCRISPR v2 or lentiCRISPR v2 containing sgKRAS-WT, sgKRAS-G12V, or sgKRAS-G12D), 0.25 μg of pMD2.G (Addgene, USA), 0.75 μg of psPAX2 (Addgene, USA) and 7 μl of Lipofectamine™ 3000 (Life Technologies, USA). The medium was changed after 6 hours of incubation at 37 °C and 5% CO_2_. The first and second viral supernatants were collected 24 and 52 hours after transfection, respectively. Harvested viral supernatants were filtered through a 0.22-μm membrane and stored at −80 °C. The lentiviral titrations were carried out using a Lenti-X™ qRT-PCR Titration Kit (Clontech, USA). For all lentiviruses used in this study, HEK293T cells were used as a standard to measure plaque forming unit (PFU), and were used to determine the multiplicity of infection as 1.

### Lentivirus transduction

To evaluate the effect of targeting *KRAS* by sgRNAs, HEK293T, SW480, SW620, SNU407, and AsPC-1 cells were transduced with the harvested lentiviral particles containing Cas9 in the absence or presence of the sgRNAs as indicated. Briefly, approximately 1–2 × 10^4^ cells were seeded in a 24-well plate at 30% confluency. Each cell line was then separately transduced in the presence of 8 μg/mL of polybrene (Sigma-Aldrich, USA) with lentiviral particles encoding Cas9 alone or in combination with sgKRAS-WT, sgKRAS-G12V, or sgKRAS-G12D. The medium was changed one day after the lentiviral transduction. Experiments aimed at improving efficiency of transduction utilized puromycin selections. Lentiviral transductions were carried out as described above. Three and six days after transduction, the medium changes included 1 μg/mL of puromycin.

### Cell proliferation assay

Transduced cell viability was evaluated using the MTT (Sigma-Aldrich, USA) cell proliferation assay. MTT (2 mg/mL) was added to each well of the 24-well plates, 2 days following the post-transduction medium change in the absence of puromycin or 7 days following the initiation of puromycin selection. After a 4-hour incubation at 37 °C, the supernatant was aspirated, and stained cells were dissolved in 200 μl of dimethyl sulfoxide (Sigma-Aldrich, USA). Absorbance at 540 nm was then measured using a Tecan Infinite**®** M100 PRO microtiter plate reader.

### Next-generation sequencing and mutation analysis

Genomic DNA was isolated using the DNeasy Blood & Tissue Kit (QIAGEN). Target loci were PCR-amplified from the genomic DNA of each cell line using the following primers: KRAS forward, 5′-TTCTTAAGCGTCGATGGAGG-3′; KRAS reverse, 5′- CTCATGAAAATGGTCAGAGAAACC-3′. The PCR amplification was performed using the following protocol: 5 min at 95 °C; 35 cycles of (30 s at 95 °C, 30 s at 60 °C, 30 s at 72 °C); 5 min at 72 °C. Following separation on a 2% agarose gel, size-selected products were purified using QIAquick Gel Extraction Kit (QIAGEN, USA). DNA libraries were prepared using SPARK™ DNA Sample Prep Kit (Enzymatics, USA). All samples were sequenced on a Hiseq4000 or a Nextseq sequencing system (Illumina, USA). Sequencing data was processed using AdapterRemoval, BWA, Samtools, and mpileup with default parameters. Indels located 3 bp upstream of the PAM sequence were considered to be induced by Cas9. The indel frequencies and patterns were calculated by in-house python programs and Cas-Analyzer^29^.

### Immunoblotting

Cultured cells were washed with phosphate buffered saline (PBS; Gibco, USA) and lysed with lysis buffer (50 mM Tris-HCl, pH 7.5 1% NP-40, 0.1% sodium deoxycholate, 150 mM NaCl, 50 mM NaF, 1 mM sodium pyrophosphate, 1 mM EDTA and protease/phosphatase inhibitors). Protein concentrations of the cell lysates were quantified with a Bicinchoninic Acid Protein Assay Reagent (Pierce, Rockford, IL), according to the manufacturer’s instructions. Samples containing equal quantities of total proteins were resolved in SDS-polyacrylamide denaturing gel, transferred to nitrocellulose membranes. The membranes were incubated in blocking buffer containing 1% skim milk and 1% bovine serum albumin (BSA, Sigma-Aldrich, USA) for an hour at room temperature and probed overnight at 4 °C with primary antibodies. Antibodies against KRAS (#sc-30) and Actin (#sc-1616) were purchased from Santa Cruz Biotechnology (Dallas, TX, USA).

### Statistical analysis

All data were expressed as means ± S.E.M., and statistical analysis was conducted using Graph Pad PRISM 7. Results of all groups are expressed as mean ± S.E.M. if not otherwise indicated. Comparisons between groups were made using the one-way ANOVA for multiple groups. Statistical significance as compared to the controls is denoted with ****(P < 0.0001) in the figures and the figure legends.

### Data availability

All data generated or analyzed during this study are included in this published article or will be provided by the corresponding authors upon request.

## Electronic supplementary material

Supplementary Information
