# Supplementary material for: Selective targeting of KRAS oncogenic alleles by CRISPR/Cas9 inhibits proliferation of cancer cells
Source: Sci Rep. 2018 Aug 8;8:11879. doi: 10.1038/s41598-018-30205-2 (PMC6082849; doi:10.1038/s41598-018-30205-2)

**Selective targeting of *KRAS* oncogenic alleles by CRISPR/Cas9 inhibits proliferation of cancer cells**

Wookjae Lee, Joon Ho Lee, Soyeong Jun, Ji Hyun Lee, and Duhee Bang

**Supplemental Information**

Supplementary Table 1. Cell lines used in this study.

Supplementary Figure 1. Indel patterns in SW620, SW480 and HEK293T cells following CRISPR-Cas9 editing.

Supplementary Figure 2. Indel frequencies with sgRNAs targeting WT or mutant *KRAS* and relative frequencies regarding to cell lines and frameshifts.

Supplementary Figure 3. Indel patterns in SNU407 and AsPC-1 cells with matching sgRNAs.

Supplementary Figure 4. Cell proliferation assays of puromycin-selected transduced cancer cells.

Supplementary Figure 5. Protein immunoblotting assays of SW620 and HEK293T.

**Supplementary Table 1.** Cell lines used in this study.

| **Type of mutation** | **Name of cell line** | **Age** | **Gender** | **Type of cancer** | **Tissue** | **Morphology** | **Homogeneity** |
| --- | --- | --- | --- | --- | --- | --- | --- |
| G12V | SW620 | 51 | Male | Colorectal adenocarcinoma | Colon | Epithelial | Homogeneous |
|  | SW480 | 50 | Male | Colorectal adenocarcinoma | Colon | Epithelial | Homogeneous |
| G12D | AsPC-1 | 61 | Female | Pancreatic ductal adenocarcinomas | Pancreas | Not available | Homogeneous |
|  | SNU407 | 37 | Male | Colorectal adenocarcinoma | Colon | Epithelial | Heterogeneous |

**Supplementary Figure 1**. **Indel patterns in SW620, SW480 and HEK293T cells following CRISPR-Cas9 editing.**

DNA sequencing identified major indel patterns in the *KRAS* genes of SW480 (a) and HEK293T (b) cells transduced with lentiviral vectors harboring sgKRAS-G12V and sgKRAS-WT, respectively. Blue: sgRNA target sequence; bold blue: KRAS G12 single base missense mutation; orange: PAM sequence; red: mismatched nucleotides. Relative indel percentages of SW620 cells with sgKRAS-G12V (c), SW480 cells with sgKRAS-G12V (d), and HEK293T with sgKRAS-WT (e).


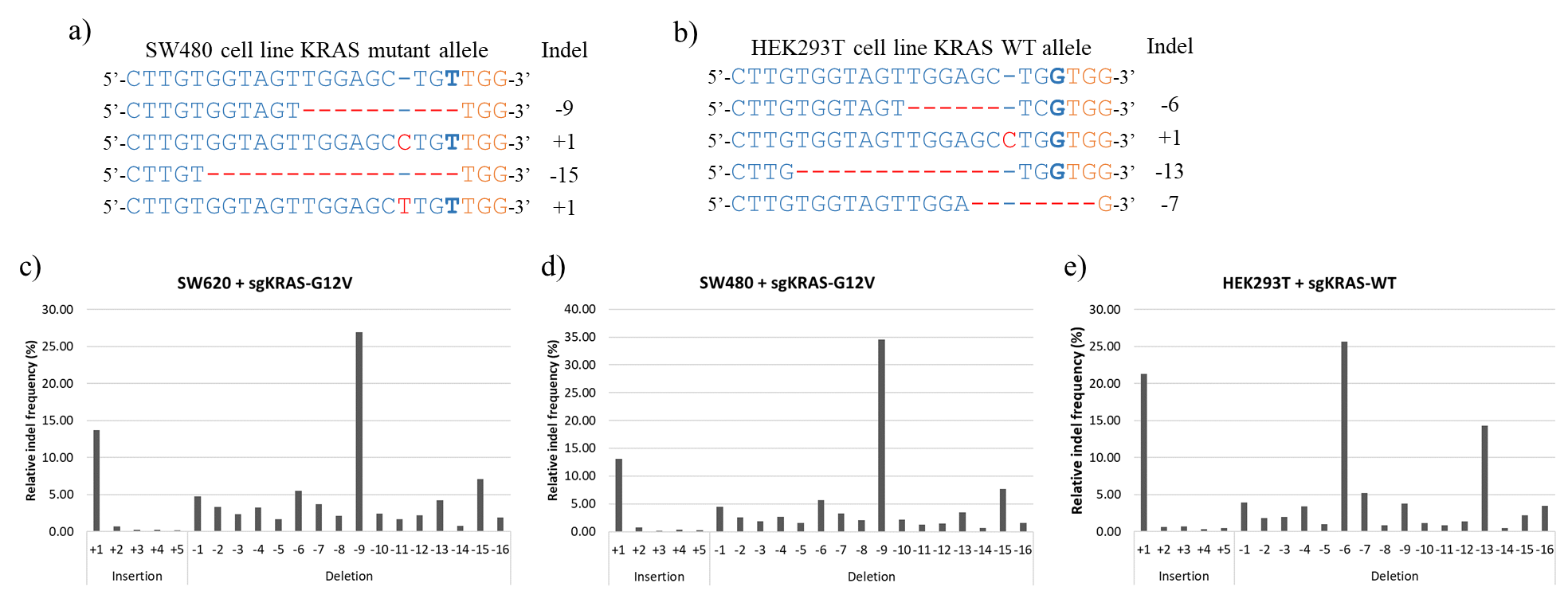


**Supplementary Figure 2. Indel frequencies with sgRNAs targeting WT or mutant *KRAS* and relative frequencies regarding to cell lines and frameshifts.**

(a) Indel frequencies showed specificities of sgRNAs regarding to cell lines. Bars represent the mean ± S.E.M.. (b) Relative frequencies of frameshifts were observed from specific sgRNAs and their matching cell lines.

**
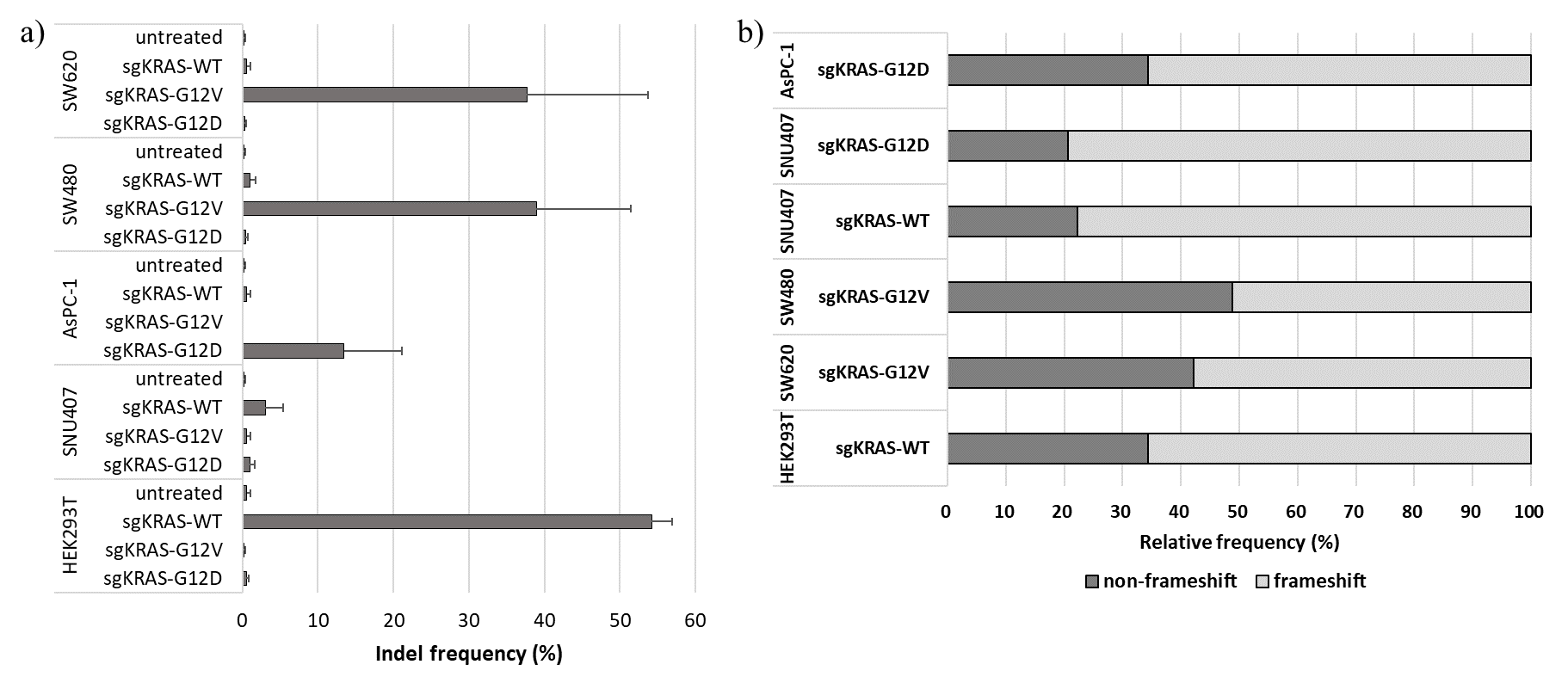
**

**Supplementary Figure 3. Indel patterns in SNU407 and AsPC-1 cells with matching sgRNAs.** Relative indel percentages of SNU407 cells with sgKRAS-WT and sgKRAS-G12D (a), and AsPC-1 cells with sgKRAS-G12D (b).

**
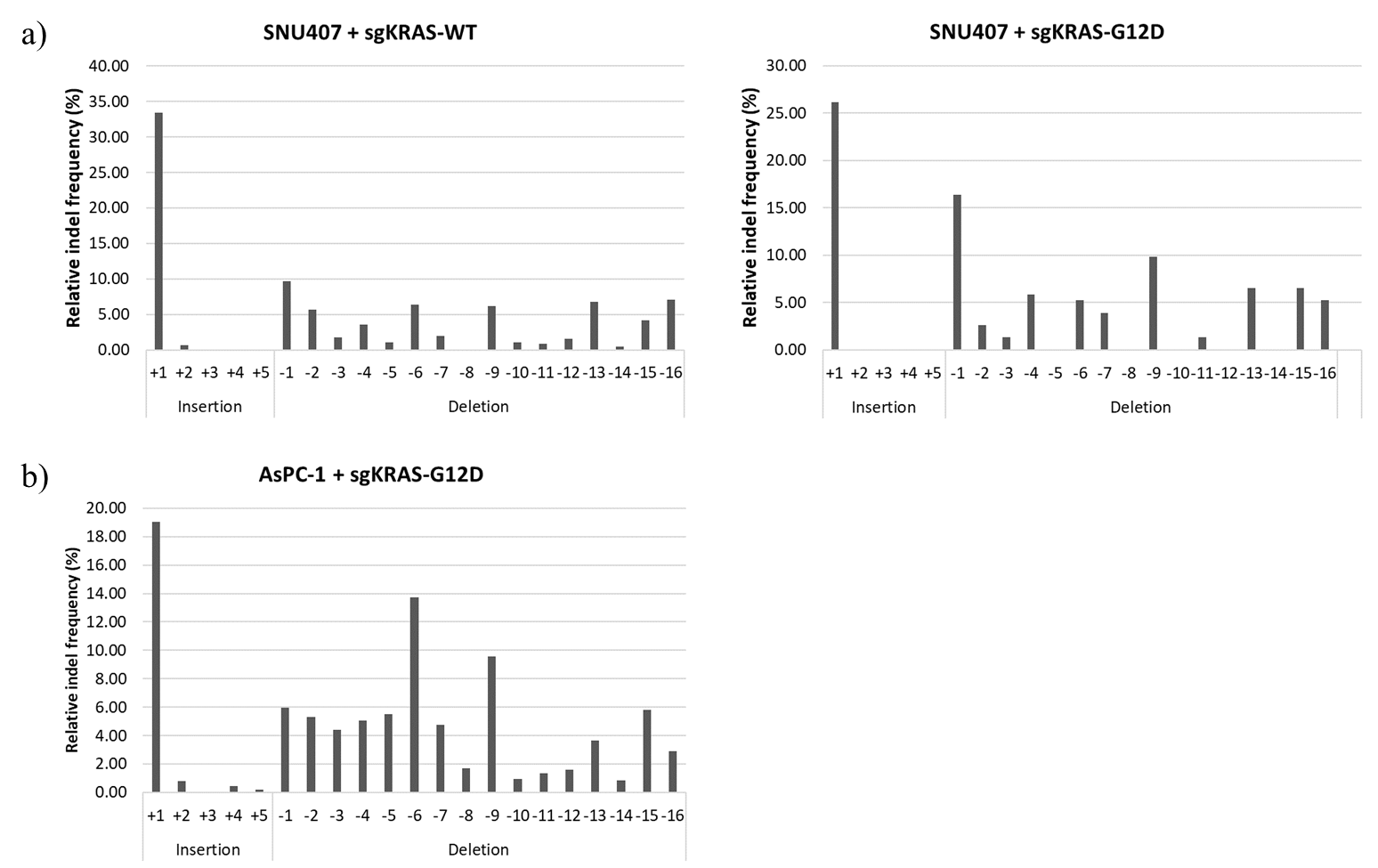
**

**Supplementary Figure 4. Cell proliferation assays of puromycin-selected transduced cancer cells.** Cell viability of puromycin-selected SW620 (a) and SW480 (b) cells transduced with lentiviral vectors encoding Cas9 alone, Cas9 and sgKRAS-G12V, or Cas9 and sgKRAS-WT. sgKRAS-G12V inhibited growth of SW620 cells to 0.95% and of SW480 cells to 0.51%. Red: sgRNA sample relevant to tested cell line. Bars represent the mean ± S.E.M. **** (P < 0.0001).


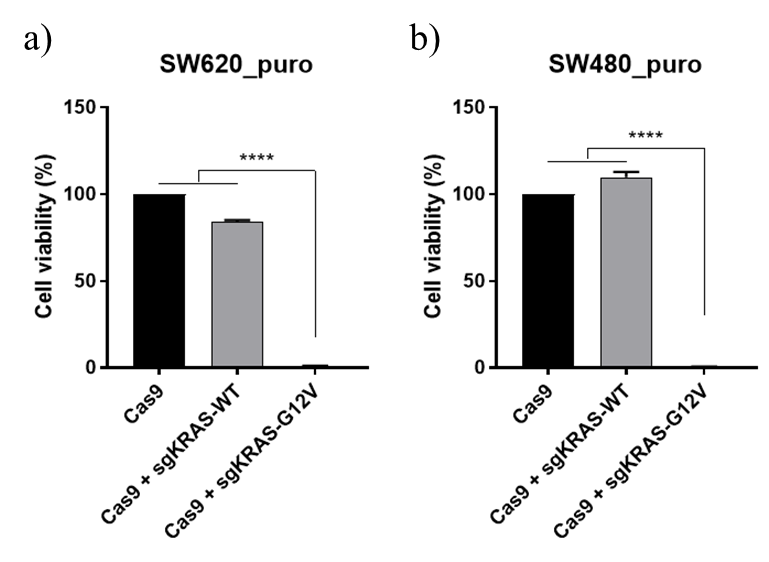


**Supplementary Figure 5. Protein immunoblotting assays of SW620 and HEK293T.**

Immunoblotting assays were performed to KRAS and Actin from SW620 and HEK293T.


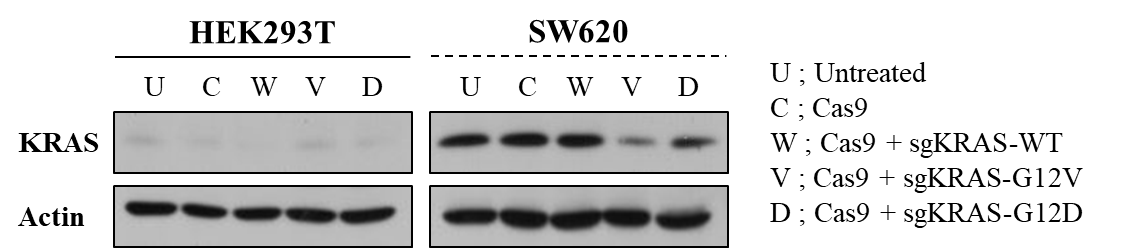

Supplement: Supplementary file 1 — Supplementary Information [file 41598_2018_30205_MOESM1_ESM.doc]
